# A low cortisol response to stress is associated with musculoskeletal pain combined with increased pain sensitivity in young adults: a longitudinal cohort study

**DOI:** 10.1186/s13075-015-0875-z

**Published:** 2015-12-10

**Authors:** Markus Paananen, Peter O’Sullivan, Leon Straker, Darren Beales, Pieter Coenen, Jaro Karppinen, Craig Pennell, Anne Smith

**Affiliations:** Centre for Life Course Epidemiology, and Medical Research Centre Oulu, Oulu University Hospital and University of Oulu, Oulu, Finland; School of Physiotherapy and Exercise Science, Curtin University, Perth, Australia; Centre of Expertise for Health and Work Ability and Disability Prevention Centre, Finnish Institute of Occupational Health, Oulu, Finland; School of Women’s and Infants’ Health, The University of Western Australia, Perth, Australia

**Keywords:** Musculoskeletal, Hypersensitivity, Quantitative sensory testing, Cortisol, Trajectory modeling

## Abstract

**Background:**

In this study, we investigated whether an abnormal hypothalamic-pituitary-adrenal (HPA) axis response to psychosocial stress at 18 years of age is associated with musculoskeletal (MS) pain alone and MS pain combined with increased pain sensitivity at 22 years of age.

**Methods:**

The study sample included 805 participants from the Western Australian Pregnancy Cohort (Raine) Study who participated in the Trier Social Stress Test (TSST) at age 18 years. Number of pain sites, pain duration, pain intensity and pain frequency were assessed at age 22 to measure severity of MS pain. Cold and pressure pain thresholds were determined at age 22. Group-based trajectory modeling was applied to establish cortisol response patterns based on the TSST. Logistic regression was used to study the association of TSST patterns with MS pain alone and MS pain combined with increased cold or pressure pain sensitivity, adjusted for relevant confounding factors. All analyses were stratified by sex.

**Results:**

The mean (standard deviation) age during the TSST was 18.3 (0.3) years, and during MS pain assessment it was 22.2 (0.6). Forty-five percent of the participants were female. Three cortisol response patterns were identified, with cluster 1 (34 % of females, 21 % of males) reflecting hyporesponse, cluster 2 (47 %, 54 %) reflecting intermediate response and cluster 3 (18 %, 24 %) reflecting hyperresponse of the HPA axis. MS pain was reported by 42 % of females and 33 % of males at age 22 years. Compared with females in cluster 2, females in cluster 1 had an increased likelihood of having any MS pain (odds ratio 2.3, 95 % confidence interval 1.0–5.0) and more severe MS pain (2.8, 1.1–6.8) if their cold pain threshold was above the median. In addition, females in cluster 1 had an increased likelihood (3.5, 1.3–9.7) of having more severe MS pain if their pressure pain threshold was below the median. No statistically significant associations were observed in males.

**Conclusions:**

This study suggests that a hyporesponsive HPA axis at age 18 years is associated with MS pain at 22 years in young females with increased pain sensitivity.

## Background

Chronic musculoskeletal (MS) pain is a disabling condition that occurs without an apparent tissue-level pathology in several functional pain disorders [1]. Its prevalence is higher among females and psychologically stressed individuals [2]. Recent findings suggest a mediating role of a major stress-regulating system, the hypothalamic-pituitary-adrenal (HPA) axis, in the association of stress with pain and pain hypersensitivity [3–5]. Several associations between variations in genes related to both HPA axis and widespread MS pain have been observed, emphasizing their potential link [6].

The HPA axis is a major system involved in the maintenance of homeostasis during stress, but it may develop first hyper- and then hypoactivity in response to chronic stress [7]. There is growing evidence that relative hypocortisolism, as a marker of stress-induced HPA axis dysfunction, may in turn increase vulnerability to pain and chronic pain disorders [5, 8–13]. In an experimental study, pharmacologically induced hypocortisolism increased mechanical pain sensitivity and potentiated hyperalgesia in healthy males, suggesting that HPA axis alterations have a causal role in pain [5]. In a cross-sectional study of female twins, a blunted cortisol diurnal pattern was associated with higher perceived pain intensity during a cold pressor test [13].

A cross-sectional population-based study found that chronic multi-site MS pain was associated with lower cortisol levels and a blunted diurnal cortisol pattern [8]. However, the same authors did not find an association between HPA axis functioning and the onset of chronic multi-site MS pain 6 years later [14]. Researchers in a longitudinal study found that a flattened diurnal cortisol profile and high post-dexamethasone cortisol levels predicted the onset of chronic multi-site MS pain 15 months later among individuals with high somatization and health-seeking behaviour [10].

Relative hypocortisolism may be present already in early childhood in association with early-life stress and psychopathology [15]. Similarly, chronic MS pain is reported already in adolescence [16], implying that the disorder may also begin early in life in some individuals. However, the association between HPA axis function and MS pain is still unclear among adolescents and young adults who are generally healthy and highly sensitive to stress [17].

Therefore, the aim of this population-based study was to investigate whether HPA axis response during acute psychosocial stress at 18 years of age was associated with subsequent MS pain presence and severity at age 22 years. In particular, we were interested in analysing the association between cortisol responses with high levels of MS pain in addition to high pain sensitivity. As such, we performed two set of analyses to evaluate (1) MS pain alone and (2) MS pain combined with increased cold pain threshold (CPT) and decreased pressure pain threshold (PPT). On the basis of previous research, we hypothesized that (1) hyporeactive HPA axis at 18 years would be associated with more severe MS pain at 22 years and (2) the association would be stronger in the presence of relative cold and pressure pain hypersensitivity.

## Methods

### Study sample and data collection

The study sample was from the Western Australian Pregnancy Cohort (Raine) Study, which includes children of pregnant women enrolled in the study at or before the 18th gestation week between 1989 and 1992. The original purpose of the study was to examine the effects of frequent and repeated ultrasound scans on pregnancy outcomes. The study is described in more detail elsewhere [18]. The population-based cohort originally included 2868 children who have been comprehensively followed from birth to 22 years of age with multiple follow-up points. The inclusion criteria of the study were gestational age between 16 and 20 weeks, sufficient English proficiency and an intention to remain in Western Australia. The cohort has been shown to be a socioeconomically representative sample of the Western Australian population [19]. The Raine Study ethical approval for the study was obtained from the University of Western Australia Human Research Ethics Committee (HREC), the Curtin University HREC for the 22-year-old cohort follow-up and the Princess Margaret Hospital for Children HREC for the 17-year-old cohort follow-up. Written informed consent was obtained from all participants.

At 17 years of age, 1399 adolescents provided questionnaire data including physical activity level and depressive symptoms, and measured height and weight were also collected. At 18 years of age, 1137 individuals participated in the Trier Social Stress Test (TSST); completed a questionnaire concerning illnesses, medication use, smoking and oral contraceptive (OC) use; and had their height and weight measured. Data on MS pain and pain sensitivity were collected at 22 years of age, when 1138 cohort members answered the Örebro Musculoskeletal Pain Questionnaire and participated in clinical assessments, including quantitative sensory testing. All active cohort members were invited to participate in each follow-up. The reason for nonparticipation at each time point was primarily unwillingness to participate.

In total, 216 participants declined to have blood collected during the TSST and 116 were excluded because of inability to complete the TSST (*n* = 2), unusable cortisol samples (*n* = 3), severe menstrual pain (*n* = 1), pregnancy (*n* = 2), lactation (*n* = 2), type 1 diabetes (*n* = 4), use of exogenous steroids (*n* = 7), neuroactive medications (*n* = 22), antidepressants (*n* = 19) or other medications affecting the HPA axis (*n* = 2), non-completion of the full test (*n* = 15) or having younger siblings participating in the study (*n* = 37). After exclusions, 366 females and 439 males had complete data on plasma total cortisol, and 277 females and 280 males had data on both MS pain and cortisol. The number of individuals included in the analysis varied from 198 to 222 among females (55 missing for covariates, 22 missing for cold pain threshold and 24 missing for pressure pain threshold) and from 198 to 215 among males (65 missing for covariates, 17 missing for cold pain threshold and 11 missing for pressure pain threshold), as only individuals with complete data on the measures of interest were included in the analysis. Because this study involved three waves of follow-up (at ages 17, 18 and 22 years), most of the missing data is due to participants choosing not to participate in one or more of these follow-ups. The other cause was incomplete data on the various questionnaires or tests.

### Trier Social Stress Test

HPA axis function was evaluated by the TSST, which includes items assessing the essential features of a reliable stressor being both uncontrollable and social-evaluative [20, 21]. It is a highly standardized protocol to induce psychological stress and cortisol response [20, 22]. The subjects arrived at the hospital between 1200 h and 1600 h for the TSST. They were informed that eating and/or drinking anything other than water within 1 h before the test would affect stress hormone levels and that therefore the test was to be conducted at least 1 h after eating. Within 15 min after arrival, an anaesthetist inserted an intravenous cannula after permission for taking blood samples was obtained. During the next 45-min rest period, the participants completed a questionnaire, and their height and weight were measured. After the rest period, the participants performed a 15-min stress test that included a free speech interview and an arithmetic task in front of a non-responsive panel of two interviewers and a dummy camera. In the debriefing discussion afterwards, the goal of the study and the nature of the stressor were explained to the participants. In total, eight blood samples were taken: just preceding the test (0 min); after completing the test (15 minutes); and at 25, 35, 45, 60, 75 and 105 min.

Blood samples were collected in ethylenediaminetetraacetic acid tubes (BD Biosciences, San Jose, CA, USA) and stored on ice during the test and were immediately centrifuged, aliquoted and frozen at −80 °C after the test until analyses. The GammaCoat™ Plasma Renin Activity ^125^I cortisol radioimmunoassay kit (DiaSorin, Stillwater, MN, USA) was used for quantifying total plasma cortisol, and concentrations in micrograms per decilitre were converted to nanomoles per litre by multiplying by 27.59. All assays were performed in duplicate against a standard curve and repeated where required. The intra- and inter-assay variability was acceptable (<10 %) for all assays.

### Musculoskeletal pain

Pain status was evaluated using five items concerning pain experience from the Örebro Musculoskeletal Pain Questionnaire [23]; we did not use the full questionnaire, as it measures likelihood of developing a persistent pain problem and related disability. The participants reported whether they had current pain in the neck, left and/or right shoulder, left and/or right arm, upper and/or lower back, left and/or right leg or other site. They were asked about the duration of their pain with ten answer options varying from ‘0 days’ to ‘over 1 year’ that were scored on a corresponding 0–9 scale. They were asked about their pain intensity during the past week, average pain intensity in the past 3 months and average frequency of pain episodes during the past 3 months with ten numerical answer options from ‘no pain’ or ‘never’ to ‘pain as bad as it could be’ or ‘always’ scored on a corresponding 0–9 scale. A pain problem severity index describing the severity of pain problems and varying from 0 to 37 was calculated by adding the following scores: a sum of pain sites (0–10), pain duration (0–9), mean of two pain intensity measures (0–9) and pain frequency (0–9). Two dichotomous variables were derived by categorizing subjects as follows: (1) no pain at all versus pain at any site and (2) pain problem severity index above and below the 75th percentile.

### Quantitative sensory testing

CPT and PPT were measured in a clinical setting according to a standardized protocol [24]. CPT was assessed using a Peltier element-based thermode with a 12.5-cm^2^ probe (Modular Sensory Analyser; Somedic AB, Hörby, Sweden) applied on the skin of the dorsal wrist. The baseline temperature was 32 °C, and the cut-off minimum temperature limit was 5 °C. The rate of temperature change was set to 1 °C/second, and the stimulus was terminated when the subject pressed a button. The participants were given the following instructions: ‘Allow the temperature to drop until the moment it reaches a point where it feels uncomfortably or painfully cold, and then press the button. This means the very first onset of discomfort or pain and not the most cold that you can bear’. To measure PPT, an algometer (Somedic AB, Farsta, Sweden) with a 1-cm^2^ contact head was applied perpendicularly to the skin at four standardized test sites in the following sequence: (1) dorsal wrist (middle of the dorsal aspect of the wrist joint line), (2) leg (the muscle belly of tibialis anterior, approximately 2.5 cm lateral and 5 cm distal to the tibial tubercle), (3) cervical spine (the trapezius muscle, at the mid-point between the C7 spinous process and the lateral acromion) and (4) lumbar spine (at the erector spinae, 2 cm lateral to the L4-L5 interspinous space). The pressure was increased with a rate of 50 kPa/second, and the cut-off maximum limit was 1000 kPa. The following instructions were given: ‘The moment the pressure increases to a point where it first feels uncomfortable or painful, press and release the button. This means the very first onset of discomfort or pain and not the most pressure that you can bear’. CPT and each PPT were tested four times, and the mean threshold value of the last three measurements was calculated (at most one missing value allowed). The sex-specific median values were used as cut-off points to identify individuals with increased pain sensitivity [25]. CPT value above the median indicated increased cold pain sensitivity, and an average of PPT values at four different sites below the median indicated increased pressure pain sensitivity.

### Covariates

OC use, smoking, body mass index (BMI), physical activity level and depressive symptoms were measured at 17 or 18 years of age and selected as covariates, as each of them has been related to HPA axis function and MS pain in previous studies [26–30]. OC use (yes or no) and smoking (yes or no) were inquired about during the TSST session and dichotomized into non-users vs. users and current smokers vs. non-smokers. BMI was measured and calculated as weight (in kilograms) divided by the square of body length (square meters) and categorized into normal (BMI <25), overweight (25 ≥ BMI < 30) and obese (BMI ≥30). If BMI at age 18 was missing, height and weight data from 17 years of age were used and BMI was categorized using the International Obesity Task Force age-specific cut-off points [31]. Participation in moderate to vigorous physical activity was measured using the short-form International Physical Activity Questionnaire [32]. As the association of physical activity with presence and severity of MS pain was U-shaped, the study subjects were categorized into three groups: (1) 0 h/week, (2) 0.1–4.9 h/week and (3) 5.0 h/week or more. The Beck Depression Inventory was used to assess depressive symptoms [33], and individuals were classified using the recommended cut-off values: minimal, 0–13; mild, 14–19; and moderate or severe, 20–63.

### Statistical analyses

The cortisol data were analysed using the group-based trajectory modeling (GBTM) approach, which is an application of the finite mixture models for longitudinal data [34]. The aim of GBTM is to identify the smallest number of latent groups following different response patterns, to which individuals are assigned probabilistically. The selection of analytical method was based on the assumption that there are distinct patterns of change in cortisol levels over time. Specifically, in light of previous findings and the allostatic load theory [4, 6], we anticipated identifying groups with atypical cortisol response (i.e., with hypo- or hyper-reactive HPA axis). Our purpose was to summarize the complex cortisol data in a parsimonious manner, as done in previous studies with GBTM using time-based cortisol data [35, 36].

As there are sex differences in pain and cortisol responses, males and females were modelled separately [17, 26, 29]. The GBTM with one to five latent groups was assessed in both sexes. The censored normal distribution was selected for the modelling after transforming the cortisol values logarithmically. To determine the number of groups that best represented the data heterogeneity, the Bayesian information criterion (BIC) was used; we also considered the practical value of the model with respect to the research question, as recommended by Nagin and Odgers [37]. The *χ*^2^ test was used to compare the covariates used in the multivariate models across the cortisol response groups.

Logistic regression analysis was applied to model the association between MS pain and cortisol response groups, with adjustments made for all potentially confounding variables and stratification by sex. Only the individuals with full data on MS pain, cortisol and covariates were included in the models. The association was first analysed using (1) pain at any site or (2) pain problem severity index above the 75th percentile as an outcome variable, with the reference group including those without or low severity of pain. In the next phase, four composite outcomes were created in light of (1) previous research in pain sensitivity testing, which does not provide clear recommended cut-off values for CPT or PPT for this age group; and (2) the need to identify those participants with the most severe cases with sufficient sample sizes in each subgroup; and (3) the need to combine pain sensitivity and pain problem severity as an outcome. The outcomes were (1) pain at any site and CPT above the median, (2) pain at any site and PPT below the median, (3) pain problem severity index above the 75th percentile and CPT above the median and (4) pain problem severity index above the 75th percentile and PPT below the median. In the analyses regarding composite variables, the reference group comprised those without pain or with low severity of pain as well as those with pain but with pain sensitivity below the median. Previously, composite outcomes have been used in the studies of, for example, cardiovascular, pulmonary and gastrointestinal diseases [38], as well as in pain research [39]. All analyses were performed using STATA 13.1 software (StataCorp, College Station, TX, USA).

## Results

The sample included 366 females and 439 males with a mean (standard deviation [SD]) age of 18.3 (0.3) years during the TSST and a mean age of 22.2 (0.6) during MS pain assessment. The distributions of variables used in the study are shown in the Table 1.

**Table 1:** Distribution of variables used in the study

	Females, *n* (%)	Males, *n* (%)
Cortisol response group		
Cluster 1	126 (34)	94 (21)
Cluster 2	173 (47)	238 (54)
Cluster 3	67 (18)	107 (24)
Oral contraceptive use		
No	227 (62)	
Yes	139 (38)	
Smoking		
No	324 (89)	355 (82)
Yes	41 (11)	79 (18)
Body mass index		
Normal	261 (72)	313 (72)
Overweight	58 (16)	79 (18)
Obese	43 (12)	41 (10)
Physical activity level		
0 h/wk	139 (45)	108 (32)
0.1–4.9 h/wk	112 (37)	114 (34)
≥ 5.0 h/wk	56 (18)	117 (35)
Depressive symptoms		
Minimal	219 (72)	309 (86)
Mild	42 (14)	33 (9)
Moderate to severe	42 (14)	16 (5)
MS pain		
No	160 (58)	188 (67)
Yes	117 (42)	92 (33)
MS pain and CPT > P50^a^		
No	193 (77)	218 (85)
Yes	57 (23)	37 (15)
MS pain and PPT < P50^b^		
No	189 (76)	227 (86)
Yes	59 (24)	37 (14)
Pain problem severity index^c^		
Low	204 (74)	210 (75)
High^c^	73 (26)	70 (25)
High pain^c^ and CPT > P50^a^		
No	215 (86)	228 (89)
Yes	35 (14)	27 (11)
High pain^c^ and PPT < P50^b^		
No	219 (88)	235 (89)
Yes	29 (12)	29 (11)

*MS* musculoskeletal, *CPT* cold pain threshold, *PPT* pressure pain threshold

^a^Median (P50) CPT = 15 °C in females and 7 °C in males

^b^Median (P50) PPT = 318 kPa in females and 419 kPa in males

^c^High pain problem severity index = 15 or more in females and 9 or more in males

### Musculoskeletal pain

MS pain at any site was reported by 42 % of females and 33 % of males (Table 1). The pain problem severity indexes ranged from 0 to 33 among females and from 0 to 29 among males, and the mean scores were 17 (SD 7) among females and 13 (SD 6) among males having any MS pain. Females with a score of 15 or more belonged to the highest quartile, whereas for males the 75th percentile was 9, hereafter referred to as *high pain problem severity index*. CPT was above the median (females 15 °C or more, males 7 °C or more) in 54 % of females and 48 % of males with any pain, and in 59 % of females and 48 % of males with a high pain problem severity index. PPT below the median (females 318 kPa or less, males 419 kPa or less) was found in 54 % of females and 45 % of males with any pain and in 49 % of females and 47 % of males with a high pain problem severity index.

### Cortisol response groups

The three-group model was considered to fit the data best in both sexes. It showed substantially lower BIC values (i.e., a better fit) than the models with one or two groups and the sizes of groups were reasonable providing meaningful interpretation of the data. Each individual had a high probability of belonging to their allocated group, confirming model adequacy, with average posterior probabilities being 0.97 for cluster 1, 0.97 for cluster 2, and 0.97 for cluster 3 among females, and 0.96, 0.97, and 0.96 among males, respectively.

**Fig. 1:**
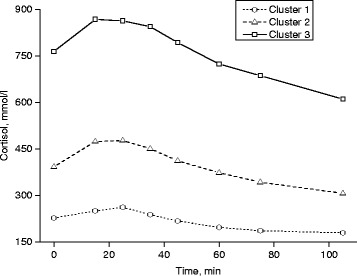
Actual cortisol values in different cortisol response groups in females

**Fig. 2:**
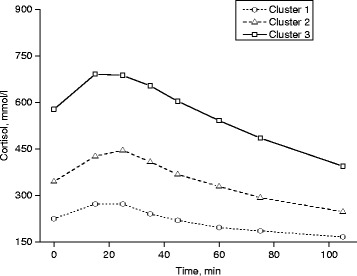
Actual cortisol values in different cortisol response groups in males

The mean baseline, peak and minimum cortisol values over the study period were the highest in cluster 3 (764/947/584 mmol/L, respectively, in females; 577/760/373 mmol/L, respectively, in males), followed by cluster 2 (392/523/280 mmol/L, respectively; 344/485/233 mmol/L, respectively) and cluster 1 (227/295/152 mmol/L, respectively; 225/301/150 mmol/L, respectively). Cluster 1, including 34 % of females and 21 % of males, had only a slight change in cortisol, with small differences between baseline and peak (difference between baseline and peak 68 mmol/L in females, 76 mmol/L in males) (Figs. 1 and 2). Cluster 2, including 47 % of females and 54 % of males, showed an immediate cortisol response followed by a consistent return to baseline, and peak values differed clearly from baseline (128 mmol/L, 140 mmol/L). Cluster 3, including 18 % of females and 24 % of males, displayed a substantial response with a large variation in cortisol values between baseline and peak (178 mmol/L, 183 mmol/L).

Cluster 3 included significantly more females using OC (87 %) than cluster 2 (39 %) or cluster 1 (11 %) (*p* < 0.001) (Table 2). Males with a high physical activity level were underrepresented in cluster 3 (*p* = 0.048), and a similar but statistically non-significant trend was seen among females (*p* = 0.090). Obesity (*p* = 0.073) and smoking (*p* = 0.085) tended to be associated with being in cluster 1 among females, but these associations were not statistically significant. Also, there were no significant differences in depressive symptoms across the cortisol response groups.

**Table 2:** Descriptive statistics for covariates according to cortisol response groups

	Females, *n* (%)				Males, *n* (%)			
	Cluster 1	Cluster 2	Cluster 3	*p* Value^a^	Cluster 1	Cluster 2	Cluster 3	*p* Value^a^
Oral contraceptive use				<0.001				
No	112 (89)	106 (61)	9 (13)					
Yes	14 (11)	67 (39)	58 (87)					
Smoking				0.085				0.300
No	107 (85)	153 (89)	64 (96)		74 (79)	190 (81)	91 (87)	
Yes	19 (15)	19 (11)	3 (5)		20 (21)	45 (19)	14 (13)	
Body mass index				0.073				0.955
Normal	83 (67)	128 (74)	50 (76)		67 (73)	171 (73)	75 (71)	
Overweight	18 (15)	28 (16)	12 (18)		16 (17)	41 (18)	22 (21)	
Obese	23 (19)	16 (9)	4 (6)		9 (10)	23 (10)	9 (9)	
Physical activity level				0.090				0.048
0 h/wk	50 (48)	57 (40)	32 (53)		26 (38)	49 (27)	33 (38)	
0.1–4.9 h/wk	31 (30)	57 (40)	24 (39)		18 (26)	63 (34)	33 (38)	
≥ 5.0 h/wk	23 (22)	28 (20)	5 (8)		25 (36)	72 (39)	20 (23)	
Depressive symptoms				0.393				0.167
Minimal	73 (70)	103 (73)	43 (75)		68 (91)	171 (86)	70 (82)	
Mild	12 (11)	22 (16)	8 (14)		2 (3)	20 (10)	11 (13)	
Moderate to severe	20 (19)	16 (11)	6 (11)		5 (7)	7 (4)	4 (5)	

^a^ *p* Values derived from *χ* ^2^ test

### Association between cortisol response and MS pain

Compared with cluster 2, belonging to cluster 1 or cluster 3 (i.e., cortisol hypo- or hyper-responsiveness) was not associated with any MS pain or a high pain problem severity index in either females or males (Tables 3 and 4). The females in cluster 1 demonstrated a significant association with any MS pain (odds ratio 2.3, 95 % confidence interval 1.0–5.0) and with high pain problem severity index (2.8, 1.1–6.8) if CPT was above the median. The females in cluster 1 also had an increased likelihood of a high pain problem severity index if PPT was below the median (3.5, 1.3–9.7), whereas any MS pain in combination with PPT below the median showed only a borderline significant association (2.2, 1.0–4.8). Among males, all associations remained statistically non-significant (Table 4).

**Table 3:** Multivariate logistic regression analysis for variables associated with musculoskeletal pain alone and combined with increased pain sensitivity among females

	MS pain only		MS pain combined with CPT > P50^a^		MS pain combined with PPT < P50^a^	
	Any pain	High pain problem severity^b^	Any pain	High pain problem severity^b^	Any pain	High pain problem severity^b^
Cortisol response group						
Cluster 1	1.5 (0.8–2.8)	1.8 (0.9–3.7)	2.3 (1.0–5.0)^c^	2.8 (1.1–6.8)^c^	2.2 (1.0–4.8)	3.5 (1.3–9.7)^c^
Cluster 2	1.0	1.0	1.0	1.0	1.0	1.0
Cluster 3	0.6 (0.3–1.4)	1.1 (0.4–2.8)	0.9 (0.3–2.7)	1.5 (0.4–5.5)	0.8 (0.3–2.6)	1.7 (0.3–8.2)
Oral contraceptive use						
No	1.0	1.0	1.0	1.0	1.0	1.0
Yes	1.3 (0.7–2.5)	1.0 (0.5–2.1)	0.8 (0.4–2.0)	0.6 (0.2–1.8)	0.8 (0.3–1.9)	0.4 (0.1–1.4)
Smoking						
No	1.0	1.0	1.0	1.0	1.0	1.0
Yes	0.9 (0.3–2.9)	1.6 (0.5–5.3)	0.7 (0.1–3.6)	1.1 (0.2–6.2)	0.2 (0.0–1.9)	0.7 (0.1–6.7)
Body mass index						
Normal	1.0	1.0	1.0	1.0	1.0	1.0
Overweight	1.2 (0.6–2.6)	1.3 (0.6–2.9)	0.6 (0.2–1.6)	0.7 (0.2–2.2)	0.4 (0.1–1.3)	0.9 (0.3–3.1)
Obese	1.3 (0.4–3.6)	0.6 (0.2–1.9)	0.7 (0.2–2.5)	0.4 (0.1–2.1)	0.6 (0.2–2.3)	0.2 (0.0–2.0)
Physical activity level						
0 h/wk	1.0	1.0	1.0	1.0	1.0	1.0
0.1–4.9 h/wk	1.1 (0.6–2.1)	1.3 (0.6–2.5)	1.0 (0.5–2.1)	1.1 (0.4–2.5)	0.7 (0.3–1.5)	0.6 (0.2–1.7)
≥ 5.0 h/wk	0.8 (0.4–1.6)	0.8 (0.3–2.0)	0.5 (0.2–1.3)	0.7 (0.2–2.0)	0.3 (0.1–0.8)	0.3 (0.1–1.2)
Depressive symptoms						
Minimal	1.0	1.0	1.0	1.0	1.0	1.0
Mild	1.8 (0.9–3.9)	1.7 (0.7–3.7)	2.0 (0.8–4.8)	1.9 (0.7–5.2)	2.0 (0.8–5.0)	1.0 (0.3–3.5)
Moderate to severe	1.3 (0.5–3.0)	1.7 (0.7–4.3)	0.7 (0.2–2.1)	0.8 (0.2–2.9)	0.9 (0.3–2.6)	0.6 (0.2–2.5)

*MS* musculoskeletal, *CPT* cold pain threshold, *PPT* pressure pain threshold

Data are presented as odds ratios with 95 % confidence intervals

^a^Median (P50) CPT = 15 °C; median PPT = 318 kPa

^b^High pain problem severity index = 15 or more

^c^ *p* < 0.05

All covariates were included in the multivariate model. Reference group includes those without pain or with a low severity of pain and those with pain but with CPT < P50 or PPT > P50

The number of individuals included in the analysis varied from 198 to 222 due to missing data

**Table 4:** Multivariate logistic regression analysis for variables associated with musculoskeletal pain alone and combined with increased pain sensitivity among males

	MS pain only		MS pain combined with CPT > P50^a^		MS pain combined with PPT < P50^a^	
	Any pain	High pain problem severity^b^	Any pain	High pain problem severity^b^	Any pain	High pain problem severity^b^
Cortisol response group						
Cluster 1	0.7 (0.3–1.5)	0.6 (0.3–1.5)	1.2 (0.4–3.3)	1.6 (0.5–4.9)	1.5 (0.6–4.1)	1.7 (0.6–4.9)
Cluster 2	1.0	1.0	1.0	1.0	1.0	1.0
Cluster 3	0.9 (0.4–1.8)	0.8 (0.3–1.8)	1.0 (0.4–2.9)	1.2 (0.3–3.9)	2.1 (0.9–5.4)	2.1 (0.7–5.9)
Smoking						
No	1.0	1.0	1.0	1.0	1.0	1.0
Yes	1.1 (0.4–2.8)	1.7 (0.6–4.4)	0.2 (0.0–1.9)	0.3 (0.0–2.9)	0.6 (0.1–2.8)	0.7 (0.2–3.7)
Body mass index						
Normal	1.0	1.0	1.0	1.0	1.0	1.0
Overweight	1.5 (0.7–3.0)	1.8 (0.8–3.8)	0.9 (0.3–2.6)	1.1 (0.4–3.6)	0.7 (0.3–2.0)	1.0 (0.3–3.1)
Obese	1.7 (0.5–6.2)	2.9 (0.8–10.8)	1.1 (0.1–9.5)	1.6 (0.2–15.8)	1.5 (0.3–7.7)	2.2 (0.4–11.7)
Physical activity level						
0 h/wk	1.0	1.0	1.0	1.0	1.0	1.0
0.1–4.9 h/wk	0.7 (0.3–1.4)	0.4 (0.2–1.0)	0.4 (0.1–1.3)	0.3 (0.1–1.2)	0.8 (0.3–2.1)	0.6 (0.2–1.8)
≥ 5.0 h/wk	1.0 (0.5–2.1)	0.8 (0.4–1.8)	0.8 (0.3–2.0)	0.8 (0.3–2.4)	0.8 (0.3–2.1)	0.9 (0.3–2.5)
Depressive symptoms						
Minimal	1.0	1.0	1.0	1.0	1.0	1.0
Mild	1.4 (0.5–4.2)	1.6 (0.5–4.9)	3.1 (0.9–10.8)	3.3 (0.9–12.8)	1.4 (0.3–5.5)	1.8 (0.4–7.5)
Moderate to severe	4.2 (1.0–17.9)	3.7 (0.9–15.3)	1.2 (0.1–10.9)	1.5 (0.2–15.2)	0.7 (0.1–6.4)	0.9 (0.1–8.4)

*MS* musculoskeletal, *CPT* cold pain threshold, *PPT* pressure pain threshold

Data are presented as odds ratios with 95 % confidence intervals

^a^Median (P50) CPT = 7 °C; median PPT = 419 kPa

^b^High pain problem severity index = 9 or more

All covariates were included in the multivariate model. Reference group includes those without pain or with a low severity of pain and those with pain but with CPT < P50 or PPT > P50

The number of individuals included in the analysis varied from 198 to 215 due to missing data

## Discussion

In this study, in females with pain sensitivity measures above the 50th percentile, a hypoactive HPA axis at age 18 years was associated with both the presence and the severity of pain problems at 22 years. This was evidenced by cluster 1 displaying higher odds than cluster 2 for pain presence and pain problem severity in those with higher cold pain sensitivity, as well as higher odds for pain problems in those with higher pressure pain sensitivity. The associations were independent of the effects of OC use, smoking, BMI, physical activity level and depressive symptoms.

Our study is the first analysis of the association between HPA axis stress response and MS pain in young adults. It was conducted in a representative birth cohort and had a longitudinal design with a 4-year follow-up. Cortisol stress response was measured under controlled conditions using a previously established protocol [20]. Cortisol responses to laboratory stress correlated strongly with total cortisol over the day, but not with cortisol awakening response or cortisol slope in a recent study [40]. In this respect, we believe that our cortisol response groups, although based on response during short-term stimuli, reflected the volume of cortisol secretion in repeated real-life stress.

Using GBTM, we identified three groups showing different cortisol stress responses in young males and females. In a recent study, researchers applied the same statistical method with four saliva cortisol samples collected from a group of adolescent females during the TSST [34]. They found normative (59 %), hyporesponsive (27 %) and hyperresponsive (15 %) groups, which corresponds well with our results. Van Ryzin et al. used GBTM with diurnal cortisol data among preschool children [35] and also found two atypical cortisol patterns (i.e., a hyper-cortisol group [10 %] and a hypo-cortisol group [16 %]) in addition to a normative group (74 %). In the majority of TSST studies, investigators have used raw cortisol values or calculated area under curve parameters, and have applied the traditional statistical techniques [11, 12]. These methods assume sample homogeneity with respect to change over time, whereas underlying subgroups representing different patterns of change can be captured with the group-based trajectory approach [37].

Our findings are broadly in line with previous studies linking HPA axis dysfunction with MS pain [8–12] and increased pain sensitivity [5, 13]. To our knowledge, there are only a few previous studies on the relationship between cortisol response in the TSST and MS pain [11, 12]. One study showed decreased salivary cortisol levels after awakening but not during the TSST among adolescents with overtiredness, dizziness and MS pain [12]. However, the association of MS pain and cortisol levels was not analysed separately from other symptoms, and the TSST was preceded by other potentially stressful tasks. In a small-scale study, middle-aged patients with fibromyalgia showed significantly reduced plasma cortisol response during the TSST, supporting our findings [11]. A flattened diurnal cortisol profile predicted new-onset widespread pain among individuals with high somatization and health-seeking behaviour in a British study [10], and another population-based study also showed that chronic multi-site MS pain was associated with lower cortisol levels and blunted diurnal slope [8]. The researchers in these two studies did not analyse stress-related cortisol levels, however, and the findings are not directly comparable to ours. In one study, researchers did not detect an association between 24-h urinary free cortisol and functional MS symptoms [41].

Interestingly, we found that a hyporesponsive HPA axis was significantly associated with MS pain in females only in combination with increased pain sensitivity. A population-based sample of young adults with MS pain is likely to be heterogeneous, with some having injury- or disease-related pain and others with more non-specific symptoms. Relative hypocortisolism may explain pain in certain subgroups by enhancing both central and peripheral sensitization of pain pathways by increased pro-inflammatory activity [42, 43]. Among patients with back pain, increased levels of pro-inflammatory cytokines together with a hyporesponsive HPA axis predicted failure in surgery [9], whereas patients with chronic widespread pain showed lower levels of Th2 anti-inflammatory cytokines than controls [44]. Pro-inflammatory cytokines may activate’ a sickness response’, which is believed to explain typical symptoms in functional pain syndromes, including exaggerated pain sensitivity [43]. This potential underlying mechanism supports our results, assuming that pain hypersensitivity is a marker of sensitization of the nervous system.

We did not find any association between HPA axis function and MS pain in males. Previous studies have reported that, compared with males, females have more chronic pain disorders [1], a lower pain threshold [25] and a lower cortisol response to stress [45]. In our study, cluster 1 had a higher proportion of females (34 %) than males (21 %), and females showed lower cortisol values during the TSST than males after controlling for OC use (data not shown). This lower cortisol response, as a sign of relative hypocortisolism, observed in females more often than males may contribute to the higher prevalence of pain disorders in females [45], and also explains why we did not find a significant association in males. The sex hormones may have a role in the reported differences between sexes [45]. Oestradiol has been shown to induce HPA axis stimulation [46] and also to improve pain perception [47]. Although females generally show lower HPA axis response to stress than males of the same age [45], the response during the luteal menstrual phase (high oestrogen level) is higher than during the follicular phase (low oestrogen level) and is comparable to that of males [48]. An explanation for our findings of sex differences warrants further studies controlling for menstrual cycle phase.

An important limitation of our study is that we did not have information about menstrual cycle phase, which may potentially confound the results. Variation in oestrogen levels influencing cortisol binding capacity is likely to explain intra- and inter-individual differences among females [28], such as our finding that OC use is associated with a higher total plasma cortisol response in the TSST. We were not able to analyse females on OC separately, owing to sample size restrictions. As excluding females on OC did not change the overall results (non-reported data), however, all females were analysed together. As cortisol values were higher at the beginning than at the end of the TSST in all response groups, a start point of HPA axis activation and true baseline values cannot be determined. Although cluster 1 and 3 were interpreted as hyporesponsive and hyperresponsive, we cannot exclude that the groups reflect different baseline levels rather than differential responsiveness. Unfortunately, we did not have subjective or objective measures of stress related to anticipation or cannulation during the rest phase, nor did we have previous data on baseline cortisol levels.

Potential fluctuation of HPA axis function over an extended time remains unclear. The TSST has been performed on only one occasion in the Raine cohort. HPA axis hyporeactivity appeared stable over 12 months among women with depression, with authors of this particular study suggesting that cortisol hyporesponse may be a trait marker [49]. During childhood and adolescence, the long-term stability of HPA axis function in stress has been shown to be at least mild to moderate [50, 51], potentially supporting this view. However, while we consider that HPA axis function at 18 years of age may be related to a pro-nociceptive state that increases the potential for altered pain sensitivity and MS pain later in life, stability of HPA axis function over time may moderate this assumption. Another limitation is that we did not assess pain status or pain thresholds at baseline, leaving their course unknown. Consequently, we could not explicitly determine the causality of the observed associations (i.e., whether HPA axis dysfunction was a cause rather than a biomarker of MS pain). Future research using multiple measures of cortisol and MS pain over time is required to further test the validity of our results.

Our measure of pain problem severity was limited to five questions derived from the well-validated Örebro Musculoskeletal Pain Questionnaire, and we believe it demonstrates face validity as it includes multiple facets of a pain problem. However, we acknowledge that this measure has not previously been used or validated against other more established measures of the severity of pain conditions. We were not able to test cut-off values other than the median for pain thresholds, because sample sizes in each subgroup would have become too small. Finally, due to the large number of participants, we were restricted to capture of pain thresholds only, without using dynamic tests of pain sensitivity, which presents a somewhat limited assessment of the pain-processing system [52].

## Conclusions

We identified that a hyporesponsive HPA axis at 18 years of age is associated with MS pain at age 22 years in young adults. However, the association between cortisol stress response and MS pain was found only in females and when pain was related to increased pain sensitivity. In the future, studies with longitudinal cortisol data and MS pain would be useful to further elucidate the nature of the relationship between HPA axis function and MS pain, including the sex discrepancy.

## Abbreviations

BIC: Bayesian information criterion
BMI: body mass index
CPT: cold pain threshold
GBTM: group-based trajectory modelling
HPA: hypothalamic-pituitary-adrenal
HREC: human research ethics committee
MS: musculoskeletal
OC: oral contraceptive
PPT: pressure pain threshold
SD: standard deviation
TSST: Trier Social Stress Test
